# Chemical structure and biological properties of a polysaccharide isolated from *Pleurotus sajor-caju*

**DOI:** 10.1039/c9ra02977j

**Published:** 2019-07-02

**Authors:** Palaniappan Seedevi, Abirami Ramu Ganesan, Kannan Mohan, Vasantharaja Raguraman, Murugesan Sivakumar, Palaniappan Sivasankar, Sivakumar Loganathan, Palasundaram Rajamalar, Shanmugam Vairamani, Annaian Shanmugam

**Affiliations:** Department of Environmental Science, Periyar University Salem 636011 Tamil Nadu India seedevisalem@gmail.com +91 9629201002; Centre of Advanced Study in Marine Biology, Faculty of Marine Sciences, Annamalai University Parangipettai 608 502 Tamil Nadu India; Department of Food Science and Home Economics, School of Applied Sciences, College of Engineering, Science and Technology, Fiji National University Fiji-7222; Centre for Ocean Research, Sathyabama Institute of Science & Technology Chennai 600 119 Tamil Nadu India; Sri Vasavi College Erode Tamilnadu India

## Abstract

Herein, a polysaccharide obtained from *Pleurotus sajor-caju* was fractionated *via* anion-exchange column chromatography and purified using gel permeation column chromatography. The chemical characterization of the polysaccharide indicated that it contained 90.16% total carbohydrate, 0% protein, 12.7% ash and 5.2% moisture; on the other hand, the carbon, hydrogen and nitrogen contents were found to be 31.53, 4.28 and 3.01%, respectively. The polysaccharide has the molecular weight of 79 kDa; the chemical structure of the polysaccharide is →6)α-d-Glc^iv^(1→6)α-d-Glc^iii^(1→6)β-d-Glc^ii^(1→6)α-d-Glc^i^(1→units. The polysaccharide exhibited the DPPH radical scavenging activity of 21.67–68.35% at 10–160 μg ml^−1^, ABTS radical scavenging activity of 16.01–70.09% at 25–125 μg ml^−1^, superoxide radical scavenging activity of 24.31–73.64% at 50–250 μg ml^−1^, hydroxyl radical scavenging activity of 16.64–63.51% at 25–125 μg ml^−1^ and reducing power of 0.366–1.678% at 10–120 μg ml; further evaluation of the polysaccharide revealed its anticancer activity of 18.61–63.21% at 100–500 μg ml^−1^ concentration against the AGS human gastric carcinoma cell line. The active principle of the polysaccharide may be used in the food and pharmacological industry in the future.

## Abstract 2

Herein, a polysaccharide obtained from *Pleurotus sajor-caju* was fractionated *via* anion-exchange column chromatography and purified using gel permeation column chromatography.

## Graphical abstract

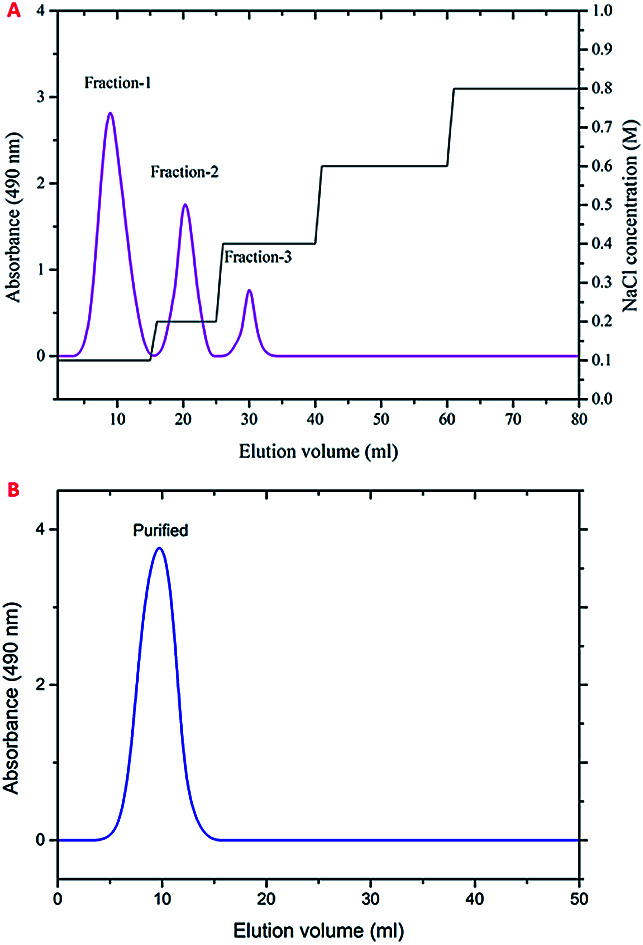

## 1. Introduction

Mushroom-derived polysaccharides are natural biological macromolecules that have gained widespread attention due to their various biological functions and nontoxic characteristics;^1,2^ in recent years, a variety of polysaccharides have been obtained from mushrooms, and their chemical structures and bioactivities, such as antioxidant, antitumor, anti-inflammatory and immunological properties, have been elucidated.^3,4^ In addition, the research findings have demonstrated that mushroom polysaccharides possess potent antioxidant activity.^5^ These antioxidant properties have been observed for polysaccharides extricated from *T. versicolor*, *L. edodes* and *Agaricus* mushrooms; moreover, these biopolymers have chelating properties that can repress lipid oxidation^6^ and are closely related to the molecular weight, monosaccharide composition, glycosidic bonds, degree of branching, and polymerization of the biopolymer.^7^ The structural features of polysaccharides obtained from mushrooms consist of β- or α- or both α- and β-linked glucan backbones displaying different patterns and degrees of branching.^8,9^

In living organisms, oxidation is a fundamental process for the production/conversion of energy for the maintenance of biological processes. However, the uncontrolled production of reactive oxygen species (ROS) is responsible for several diseases such as cancer, rheumatoid arthritis, atherosclerosis, coronary heart disorder, *etc.*^10,11^ Antioxidants are substances that can react with ROS and reduce the risk of chronic human diseases; human and other living organisms possess antioxidants that protect them from oxidative damage; however, these antioxidants are not sufficient to prevent the oxidative damage. Synthetic antioxidants, such as butylated hydroxyanisole (BHA), butylated hydroxytoluene (BHT), and propyl gallate (PG), possess strong radical scavenging activities; however, the use of these antioxidants is restricted due to their side effects.^12^ Hence, extensive interest is being devoted towards the investigation of naturally occurring antioxidants to replace synthetic antioxidants. Currently, the development of effective and safe natural antioxidants is one of the main pursuits in the field of research on antioxidants. From this perspective, the antioxidants present in mushrooms are of great interest as potential protective agents against oxidative damage.^13^ In addition, the mushroom polysaccharides have anticancer and antidiabetic activities, serve as food and are also used in the pharmaceutical industry;^14^ recently, several mushroom polysaccharides have been isolated, and their biological activities have been investigated; moreover, they have been chemically and structurally characterized.^15^ Considering the abovementioned information on mushroom polysaccharides and their biological activities, herein, an attempt was made to isolate a polysaccharide from *P. sajor-caju* and examine its chemical structure and biological properties.

## 2. Materials and methods

### 2.1. Collection and pre-treatment of the mushroom

The mushroom *P. sajor-caju* was obtained from Krishi Vigyan Kendra (KVK) – ICAR, Salem, Tamil Nadu, India. The mushroom was washed with distilled water and shade dried. The dried mushroom was cut into small pieces and powdered in a mixer grinder for further extraction.

### 2.2. Extraction of the polysaccharide

Extraction of the polysaccharide was carried out following a procedure previously described by Seedevi *et al.*^16^ Briefly, 50 g of mushroom powder was dipped in 1 : 50 volumes (2.5 l) of distilled water and kept at room temperature for 2 h; then, the mixture was homogenized and refluxed at 100 °C for 2 h. After cooling, the mixture was filtered using cheesecloth; further, the supernatant was separated from the mushroom residue by centrifugation at 6000 rpm for 20 min. The residue was further extracted thrice (three times) with double-distilled water at 100 °C for 2 h. All extracts were pooled and concentrated under reduced pressure in a rotary evaporator and dialyzed in a cellulose membrane (the molecular weight cut off 12 kDa) against distilled water for three successive days. The retained fraction was freeze-dried in a lyophilizer (Penguin plus −4 kg, Lark India) and stored in a refrigerator.

### 2.3. Deproteinization of the crude polysaccharide

The crude polysaccharide mixture was dissolved in distilled water and mixed with the Sevag reagent containing chloroform and *n*-butanol (CHCl_3_ : *n*-BuOH) (5 : 1)^17^ as previously described by Seedevi *et al.*^16^

### 2.4. Fractionation of the polysaccharide

#### 2.4.1. Ion-exchange chromatography

The crude polysaccharide was dissolved in distilled water (10 mg/10 ml), centrifuged at 6000 rpm for 10 min, and the supernatant was applied to a Q-Sepharose™ (GE-Healthcare) Fast Flow column (EX 150 mm × 50 cm) equilibrated with distilled water. The column was eluted stepwise with distilled water, with the linear gradient of 0–3 mol L^−1^ NaCl at the flow rate of 0.55 ml min^−1^, and 15 fractions were obtained. The carbohydrate content of the obtained fractions was estimated by the phenol-sulfuric acid reaction.^18^ The fractions showing higher yields were pooled together, dialyzed against distilled water and then freeze-dried.

#### 2.4.2. Purification of the fractionated polysaccharide

##### 2.4.2.1 Gel filtration chromatography

The semi-purified polysaccharide was further purified by gel permeation chromatography using the High-Resolution Sepharose 4-LB Fast Flow column (EX 100 mm × 25 cm), equilibrated with distilled water. The column was eluted stepwise with distilled water and then with 0.2 mol l^−1^ of sodium acetate buffer at the elution rate of 0.33 ml min^−1^. The carbohydrate content of the obtained fractions was estimated by the phenol-sulphuric acid reaction.^18^ The fractions showing higher yields were pooled together, dialyzed against distilled water and then freeze-dried.

### 2.5. Analysis of the biochemical composition

#### 2.5.1. Determination of total carbohydrate and protein contents

The total carbohydrate content was estimated calorimetrically by the phenol-sulfuric acid method using d-glucose as a standard.^18^ The protein content was measured by the Bradford method^19^ using bovine serum albumin as a standard.

#### 2.5.2. Determination of the ash and moisture content

The ash content of the polysaccharide was quantified gravimetrically according to the method reported by Seedevi *et al.*^20^ Typically, 0.5 g of the polysaccharide taken in a porcelain crucible was burnt at 600 °C for 8 h in a muffle furnace. The weight of the residue represents the ash content, and the results are presented as a percentage. The moisture content of the polysaccharide was determined using a micro oven; then, 0.5 mg of sample was dried at 130 °C for 2 h, and the results are presented as a percentage.

#### 2.5.3. Determination of molecular weight

The molecular weight of the polysaccharide was determined by gel permeation chromatography.^21^ The standard dextran with different molecular weights of 250, 200, 70 and 40 kDa was passed through a Sepharose 6B column, and the elution volumes were plotted against the logarithms of their respective molecular weight. The elution volume of the polysaccharide was plotted on the same graph, and the average molecular weight of the polysaccharide was determined.

#### 2.5.4. HPLC analysis of the monosaccharide composition

The crude and purified polysaccharide were dissolved in 1 ml of distilled water and mixed with an equal volume of 4.0 M trifluoroacetic acid (TFA). The sample was allowed to stand for 4 h at 100 °C, the acid-hydrolyzed sample was filtered through 0.45 μm syringe filter, and the residual acid was removed by decompression and distillation three times with methanol. The sample was injected into a high-performance liquid chromatography (HPLC) system (Rheodyne, Rohnert Park, CA, USA, Model 7010) with a 20 μl sample loop, a column (carbohydrate analysis column, 4.6 × 250 mm, Waters), and a refractive index (RI) detector. The following neutral monosaccharides were used as reference: rhamnose, xylose, mannose, galactose, fructose and glucose (Sigma).

#### 2.5.5. Spectral analysis of the polysaccharide

##### 2.5.5.1 FTIR analysis of the polysaccharide

The polysaccharide was analyzed using Bio-Rad FTIR – 40 (USA); typically, 10 mg of the polysaccharide was mixed with 100 mg of dried potassium bromide (KBr) and compressed to prepare a salt disc (10 mm diameter) to obtain its spectrum. Spectra were obtained in the wavenumber range of 4000–500 cm^−1^.

#### 2.5.6. Methylation analysis

The polysaccharide was methylated using a previously described method reported by Maity *et al.*^21^ The polysaccharide (5 mg) was kept over P_2_O_5_ in a vacuum desiccator. The dried polysaccharide and sodium hydroxide (NaOH) were dissolved in 1 ml of anhydrous DMSO followed by stirring for 30 min. The mixture was methylated with 1 ml of methyl iodide and stirred for 1 h; then, the methylated products were partitioned between CHCl_3_ and H_2_O (5 : 2, v/v) for four times. The organic layer containing the products was obtained and dried. The methylated product was hydrolysed with 90% formic acid (1 ml) at 100 °C for 1 h, and excess formic acid was evaporated by co-distillation with distilled water. After being reduced with sodium borohydride, the methylated and hydrolysed product of the polysaccharide was acetylated with pyridine-acetic anhydride (1 : 1) for 45 min at 100 °C. The alditol acetates of the methylated sugars were extracted with chloroform and analysed by GLC-MS. The GLC-MS analysis was performed *via* the Shimadzu GLC-MS Model QP-2010 Plus automatic system using a ZB-5MS capillary column (30 m × 0.25 mm). The program was isothermal at 150 °C; the hold time was 5 min, with the temperature gradient of 2°C min^−1^ up to the final temperature of 200 °C.

#### 2.5.7. ^1^H-NMR and ^13^C-NMR spectral analysis of the polysaccharide

Approximately 30 mg of the polysaccharide was dissolved in 1 ml of D_2_O (99.9%) in an NMR tube (5 mm diameter), and the ^1^H-NMR and ^13^C-NMR analyses were performed using the Varian BRUKER-500 instrument at 27 °C. The chemical shifts were expressed in parts per million (ppm).

### 2.6. Antioxidant activity

#### 2.6.1. DPPH free-radical scavenging assay

The DPPH free-radical scavenging activity of the polysaccharide was determined by following the method reported by Zhang *et al.*^22^ Briefly 0.1 mM solution of DPPH was prepared in 100% methanol, and 1 ml of this solution was added to 4 ml of sample in 40% methanol at various concentrations (10–160 μg ml^−1^). The mixture was shaken vigorously and incubated for 15 min at 30 °C in the dark. The reduction of the DPPH radical was measured by continuous monitoring of the decrease in absorption at 517 nm. Lower absorbance of the reaction mixture indicated higher free-radical scavenging activity. BHA and l-ascorbic acid were used as standards. The DPPH scavenging effect was calculated as follows:
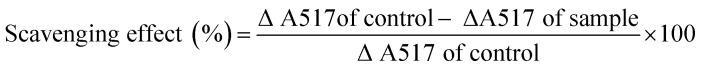


#### 2.6.2. ABTS radical scavenging assay

The radical scavenging capacity of the polysaccharide was evaluated against ABTS radical cations generated using the methodology reported by Giao *et al.*^23^ This method is based on the ability of antioxidant molecules to quench the long-lived ABTS+ species, a blue-green chromophore with a characteristic absorption at 734 nm. The addition of antioxidants to the preformed radical cation reduces it to ABTS, causing a loss of color. Results were expressed by plotting the % of inhibition, and l-ascorbic acid and BHT were used as standards.
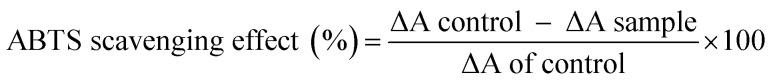


#### 2.6.3. Superoxide radical scavenging assay

The superoxide scavenging ability of the polysaccharide was assessed by the method reported by Nishikimi *et al.*^24^ The reaction mixture containing the sample (50–250 μg ml^−1^), PMS (30 mM), NADH (338 mM) and NBT (72 mM) in phosphate buffer (0.1 M pH 7.4) was incubated at room temperature for 5 minutes, and the absorbance was read at 560 nm against a blank. BHA and l-ascorbic acid were used as standards. The capability of the polysaccharide to scavenge the superoxide radical was calculated using the following equation:
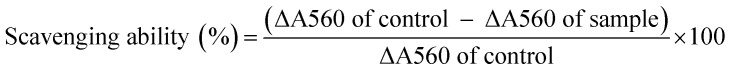


#### 2.6.4. Hydroxyl radical scavenging assay

The reaction mixture containing the sample (25–125 μg ml^−1^) was incubated with deoxyribose (3.75 mM), H_2_O_2_ (1 mM), FeCl_3_ (100 mM), EDTA (100 mM) and ascorbic acid (100 mM) in potassium phosphate buffer (20 mM, pH 7.4) for 60 minutes at 37 °C.^25^ The reaction was terminated by adding 1 ml of TBA (1%, w/v) and 1 ml of TCA (2%, w/v), and then, the tubes were heated in a boiling water bath for 15 minutes. The contents were cooled down, and the absorbance of the mixture was measured at 535 nm against the reagent blank. The decreased absorbance of the reaction mixture indicated the decreased oxidation of deoxyribose. BHA and l-ascorbic acid were used as standards.

#### 2.6.5. Reducing power

The reducing power was determined according to the method reported by Oyaizu.^26^ Each sample (10–120 μg ml^−1^) in a 2 g l^−1^ acetic acid solution (2.5 ml) was mixed with 2.5 ml of 200 mmol l^−1^ sodium phosphate buffer (pH 6.6) and 2.5 ml of 10 g l^−1^ potassium ferricyanide, and the mixture was incubated at 50 °C for 20 min. Thereafter, 2.5 ml of 100 g l^−1^ trichloroacetic acid was added, and the mixture was centrifuged at 3000 rpm for 10 min. The upper layer (5 ml) was mixed with 5 ml of deionized water and 1 ml of 1 g l^−1^ ferric chloride, and the absorbance was measured at 700 nm against a blank. BHA and l-ascorbic acid were used as standards.

### 2.7. Cytotoxicity assay

The cytotoxicity test for the polysaccharide was conducted using MTT according to the method reported by Seedevi *et al.*^20^ using Vero cells at different concentrations (100–1400 μg ml^−1^). The optical density (OD) of each well was measured using an Elisa reader at 620 nm.

#### 2.7.1. Anticancer activity

The anticancer activity of the polysaccharide against AGS human gastric carcinoma cell lines and HepG2 cell line was evaluated using the MTT assay as described by Seedevi *et al.*^20^ Vero cells were seeded (3 × 10^4^/well) in 96-well plates in 100 μl of growth medium (MEM) containing 10% FCS mixture in each well incubated at 37 °C in a 5% CO_2_ incubator. After 24 h of monolayer cell cultivation, the medium was removed and replaced by 100 μl of the polysaccharide at varying concentrations (100–500 μg ml^−1^) in the MEM medium containing 2% FCS in respective wells. The control cells were maintained in the MEM medium containing 2% FCS incubated at 37 °C in 5% CO_2_. After 72 h of incubation, 20 μl of MTT (5 mg ml^−1^) in the PBS solution/well was added followed by incubation under the abovementioned conditions for 4 h; then, the crystal formation was observed. The medium was replaced by a 100 μl of DMSO solution in each well, and the optical density (OD) of each well was measured using an Elisa reader at 620 nm.

### 2.8. Statistical analysis

All experimental data were subjected to one-way analysis of variance (ANOVA), and Dunnett's Multiple Comparison (GraphPad prism version 5.0, GraphPad Software, USA) was used to determine the difference among means at the level of 0.05.

## 3. Results

### 3.1. Fractionation, purification and yield of the polysaccharide

**Fig. 1:**
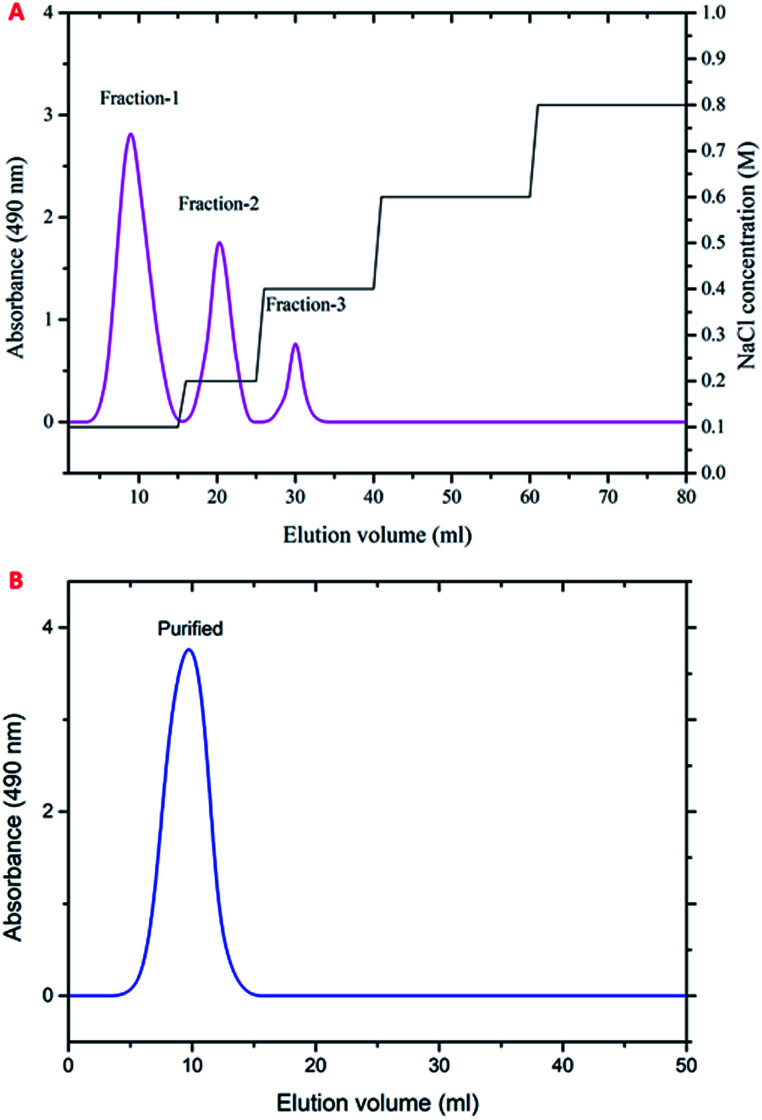
Elution profile of the polysaccharide obtained from *P. sajor-caju.* (A) Ion exchange chromatography using a Q-Sepharose column. (B) Gel filtration chromatography using a Sepharose 4-LB column.

The polysaccharide was fractionated initially *via* a Q-Sepharose column using NaCl and eluted stepwise with distilled water, with the linear gradient of 0–3 mol L^−1^ NaCl. The fractions of 0.1, 0.2 and 0.3 M/NaCl showed higher carbohydrate content and were pooled together and freeze-dried (Fig. 1A). The fractionated polysaccharide was purified through a Sepharose 4-LB column using 0.2 M sodium acetate buffer, and the fraction showed a single peak at higher carbohydrate content and was freeze-dried (Fig. 1B). The yield of the purified polysaccharide was found to be 18.01% (w/w) on a dry weight basis.

### 3.2. Chemical composition of the polysaccharide

The total carbohydrate, protein, ash and moisture contents of the polysaccharide were 90.16, 0, 12.7 and 5.2%, respectively. In the present study, the total carbohydrate content of the purified polysaccharide was higher when compared with that reported in a previous study conducted on the hot water extraction (HWE), ultrasound-assisted extraction (US) and Soxhlet extraction (SE) of a polysaccharide obtained from *Ganoderma lucidum*, which showed 66.85, 50.24 and 44.47% total carbohydrate content, respectively.^31^

### 3.3. Molecular weight of the polysaccharide

**Fig. 2:**
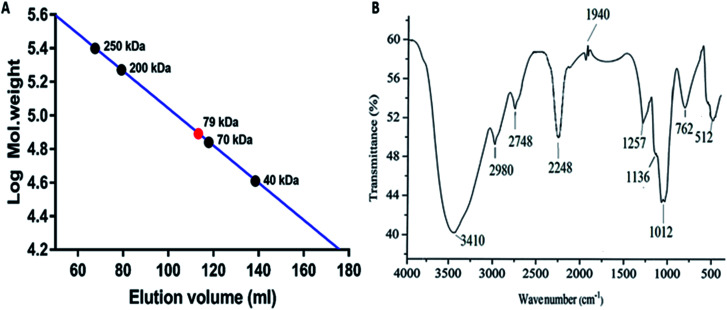
(A) Molecular weight of the polysaccharide. (B) FTIR spectrum of the polysaccharide.

The molecular weight of the polysaccharide obtained from *P. sajor-caju* was 79 kDa as compared to the standard dextran *M*_w_ 250, 200, 70 and 40 kDa (Fig. 2A). In the present study, the molecular weight was found to be higher as compared to that of the *Lentinus fusipes* mushroom polysaccharide (*M*_w_: 60 kDa).^35^

#### 3.3.1. Monosaccharide composition of the polysaccharide

The monosaccharide composition of the polysaccharide is as follows: 80.7% of glucose and 16.3% of galactose, which has been compared with that of the standard monosaccharides; this confirms that the mushroom *P. sajor-caju* polysaccharides are rich in glucans, which have been well recovered by the extraction and purification method.

#### 3.3.2. FTIR spectra of the polysaccharide

The FTIR spectra of the polysaccharide obtained from *P. sajor-caju* displayed a broad stretching peak at around 3410 cm^−1^, indicating the existence of the OH group in the molecular structure (Fig. 2B). The peak at 2980 indicated the C–H stretching vibration.^37^ The peak at 1257 cm^−1^ was assigned to the O stretching vibration of the sulfate group. The stretching vibration peaks between 1136 and 1012 cm^−1^ suggested the presence of C–O–C and C–O–H, respectively;^38^ the peak at 762 cm^−1^ indicated the presence of α-glucans.^40,41^ There was no absorbance peak around 1600 1400 cm^−1^ in the FTIR spectrum of the polysaccharide; this indicated that the protein molecules could be completely removed after deproteinization with the Sevag reagent.

#### 3.3.3. Methylation analysis

**Fig. 3:**
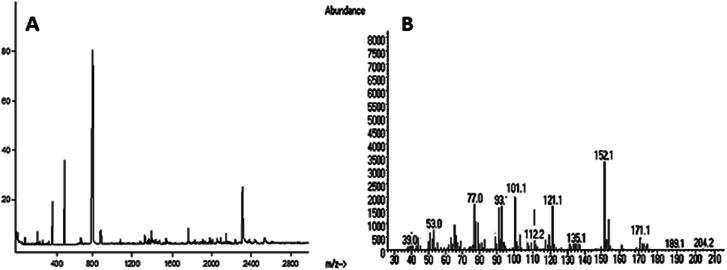
(A) Total ion chromatogram and (B) mass spectrum of the polysaccharide.

The methylation analysis of the polysaccharide showed the presence of five components (Table 1), namely 2,3,4-Me_3_-Glc, 2,3,4,-Me_3_-Glc, 2,3,4-Me_3_-Glc, 2,3,4,-Me_3_-Glc and 2,3,4,6-Me_4_-Glc in the molecular ratio of 4 : 1 : 2 : 1 : 1. The total ion and mass spectra are shown in Fig. 3A and B.

**Table 1:** GLC-MS analysis of the methylated polysaccharide

Methylated sugars	Molar ratio	Linkage-type	Major mass fragments (*m*/*z*)
2,3,4-Me_3_-Glc	4	→6)-Glcp-(1→	39, 53, 77, 101, 112, 121, 152, 171, 204
2,3,4,-Me_3_-Glc	1	→6)-Glcp-(1→	77, 101, 112, 135, 171, 189, 204
2,3,4-Me_3_-Glc	2	→6)-Glcp-(1→	39, 53, 77, 93, 101, 112, 121, 152
2,3,4,-Me_3_-Glc	1	→6)-Glcp-(1→	39, 53, 77, 101, 121, 135, 161, 171, 189
2,3,4,6-Me_4_-Glc	1	Glcp-(1→	39, 53, 77, 101, 112, 121, 135, 189, 204

#### 3.3.4. ^1^H-NMR and ^13^C-NMR spectra of the polysaccharide

**Fig. 4:**
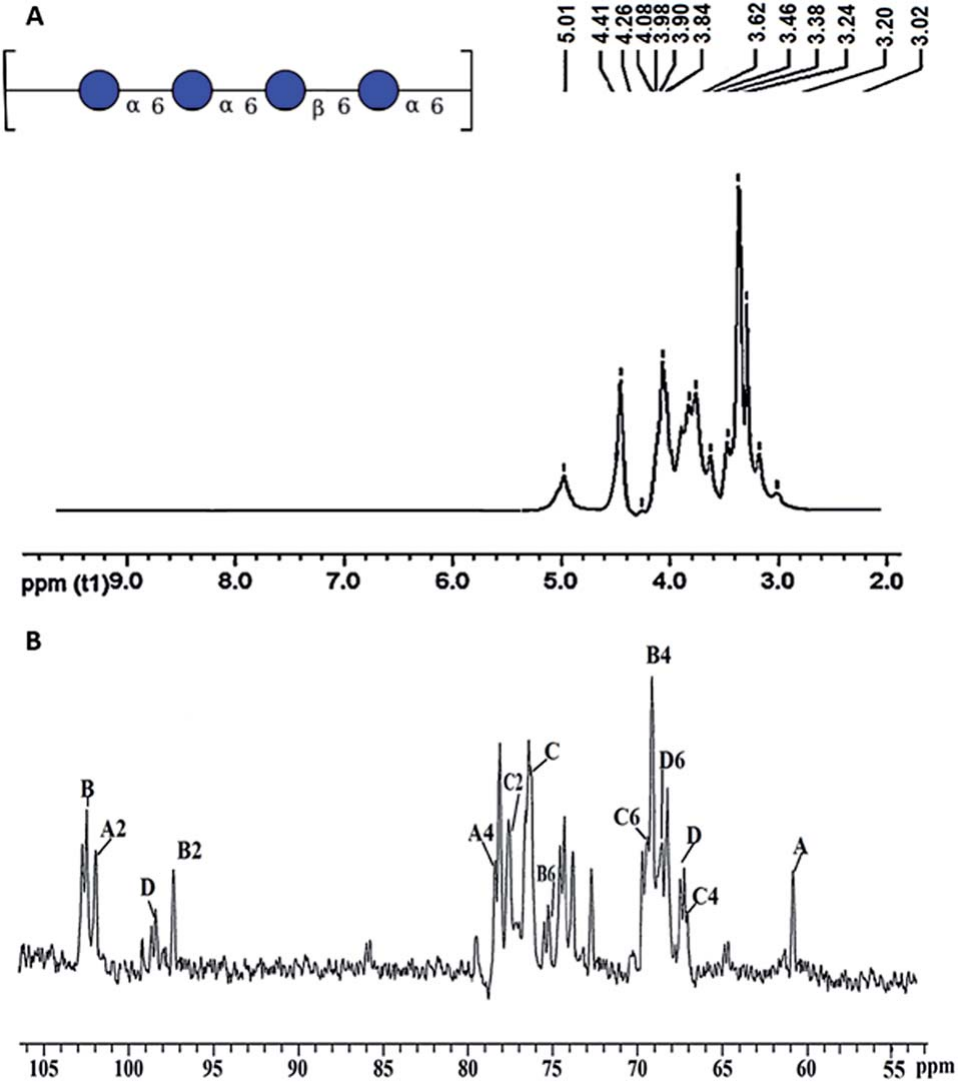
NMR spectrum of the polysaccharide: (A) ^1^H-NMR and (B) ^13^C-NMR.

The ^1^H-NMR spectra of the polysaccharide obtained from *P. sajor-caju* are depicted in Fig. 4A. The signal at *δ* 5.01 was attributed to the α-configuration,^36^ and the signal at *δ* 4.41 indicated β-configuration in the polysaccharide.^35^ The signal at *δ* 4.26–3.84 showed the α-d-glucose configuration in the polysaccharide. The signals at *δ* 3.62, 3.46 and 3.38 showed the H-5, H-5 and H-2 positions of the β-d-glucose configuration.^29^ The signal at *δ* 3.02–3.24 indicated an α-β- linkage in the glucopyranosyl residues in the polysaccharide. The ^13^C-NMR spectrum of the polysaccharide obtained from *P. sajor-caju* is depicted in Fig. 4B. The carbon chemical shifts at *δ* 101.9 (A2), *δ* 78.5 (A4) and *δ* 60.9 (A) indicate the presence of the (1,3,6)-α-d-glucopyranosyl residue.^30^ The chemical shifts at *δ* 102.5 (B), *δ* 97.4 (B2), *δ* 75.2 (B6) and *δ* 69.1 (B4) indicated (1→ 6)-β-d-glucopyranosyl (see Table 2). The chemical shifts at *δ* 77.7 (C2), *δ* 76.4 (C), *δ* 69.4 (C6) and *δ* 67.1 (C4) were due to the (1→6) linked α-d-glucopyranosyl residue. The chemical shifts at *δ* 98.5 (D), *δ* 68.8 (D6) and *δ* 67.5 (D) indicated the (1→6) linked α-d-glucopyranosyl residue.^42^ The polysaccharide linkage indicated the chemical structure of →6)α-d-Glc^iv^(1→6)α-d-Glc^iii^(1→6)β-d-Glc^ii^(1→6)α-d-Glc^i^(1→units (Table 1).

Assignment of the ^1^H and ^13^C resonances

**Table:** 

^1^H-NMR (*δ*)	Assignment
3.62	α-d-Glc^i^ – 2
3.46	α-d-Glc^i^ – 4
3.02	β-d-Glc^ii^ – 2
3.38	β-d-Glc^ii^ – 3
3.24	β-d-Glc^ii^ – 4
3.62	α-d-Glc^iii^ – 2

**Table:** 

^13^C-NMR (*δ*)	Assignment
101.9	α-d-Glc^i^ – 1
69.4	α-d-Glc^i^ – 4
68.8	α-d-Glc^i^ – 6
102.5	β-d-Glc^ii^ – 1
78.5	β-d-Glc^ii^ – 3
69.1	β-d-Glc^ii^ – 4
77.7	β-d-Glc^ii^ – 5
67.1	β-d-Glc^ii^ – 6
97.4	α-d-Glc^iii^ – 1
75.2	α-d-Glc^iii^ – 3
60.9	α-d-Glc^iii^ – 6
98.5	α-d-Glc^iv^ – 1
76.4	α-d-Glc^iv^ – 3
67.5	α-d-Glc^iv^ – 6

### 3.4. Antioxidant activity

#### 3.4.1. DPPH radical scavenging activity

**Fig. 5:**
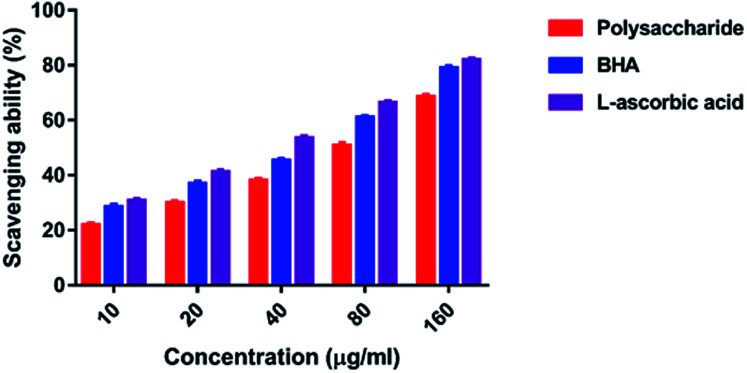
DPPH radical scavenging activity.

The DPPH free-radical assay is widely used to determine the free-radical scavenging abilities of various natural compounds due to its convenience and reproducibility.^43^ The DPPH radical scavenging activity of the polysaccharide was 21.67–68.35% at 10–160 μg ml^−1^ concentration. It was observed that an increase in the polysaccharide concentration increased the activity level. The maximum inhibition was 68.35% at 160 μg ml^−1^ concentration, and the lowest inhibition was 21.67 at 10 μg ml^−1^ concentration. However, the standard BHA and l-ascorbic acid had 78.92 and 81.76% inhibition at the highest concentration of 160 μg ml^−1^, respectively (Fig. 5).

#### 3.4.2. ABTS radical scavenging activity

**Fig. 6:**
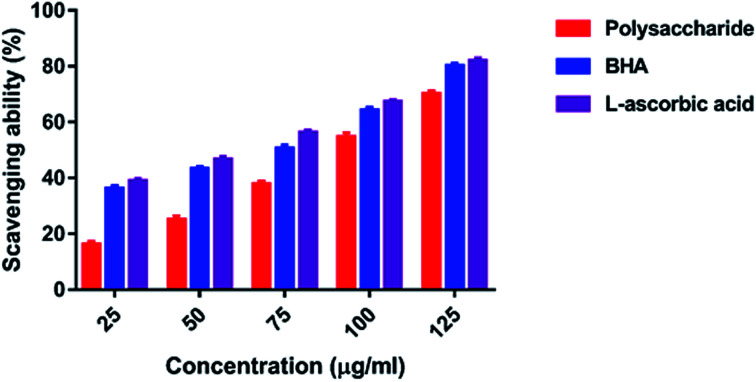
ABTS radical scavenging activity.

The ABTS radical scavenging assay is extensively applied to measure the antioxidant power of chemical components.^44^ The ABTS radical scavenging activity of the polysaccharide was 16.01–70.09% at 25–125 μg ml^−1^ concentration (Fig. 6). This activity was found to increase when the polysaccharide concentration was increased. The maximum ABTS radical scavenging activity was 70.09% at 125 μg ml^−1^, and the minimum ABTS radical scavenging activity was 16.01% at 25 μg ml^−1^ concentration. However, the standard BHA and l-ascorbic acid had 80.01 and 81.95% ABTS radical scavenging activity at the highest concentration of 125 μg ml^−1^, respectively.

#### 3.4.3. Superoxide radical scavenging activity

**Fig. 7:**
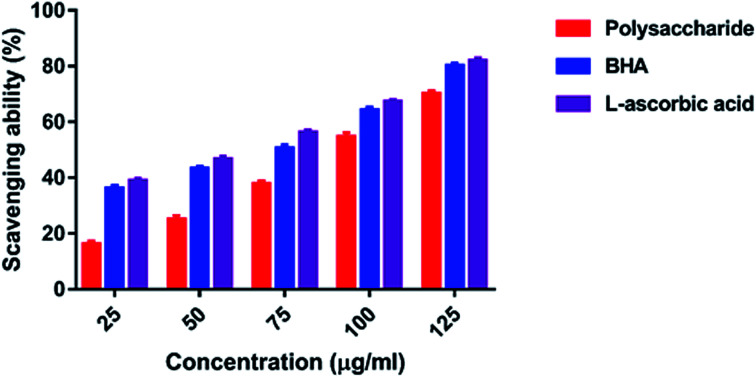
Superoxide radical scavenging activity.

The superoxide anion is also known to indirectly initiate lipid peroxidation as a result of the formation of H_2_O_2_, creating precursors of hydroxyl radicals.^45^ There was a marked inhibitory effect of polysaccharides on superoxide radicals, and it was concentration dependent. The scavenging effect of the superoxide radical was 24.31–73.64% at 50–250 μg ml^−1^ (Fig. 7). The maximum scavenging effect was 73.64% at 250 μg ml^−1^ concentration, and the lowest scavenging effect was 24.31% at 50 μg ml^−1^ concentration. On the other hand, the standard BHA and l-ascorbic acid showed the scavenging effects of 81.34 and 83.48% at 125 μg ml^−1^ concentration, respectively.

#### 3.4.4. Hydroxyl radical scavenging activity

**Fig. 8:**
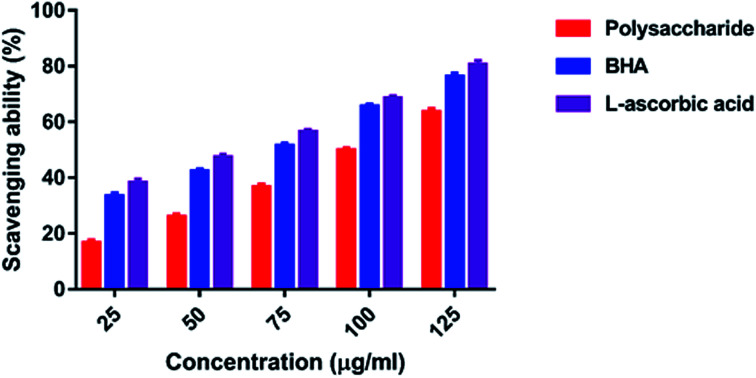
Hydroxyl radical scavenging activity.

The hydroxyl radical is very reactive and can be generated in biological cells through the Fenton reaction. The effects of polysaccharide on oxidative damage induced by hydroxyl radicals at different concentrations ranging from 25 to 125 μg ml^−1^ were found between 16.64 and 63.51% (Fig. 8). A maximum of 63.51% inhibition was observed at the highest concentration of 125 μg ml^−1^ of polysaccharide, and lowest inhibition was observed at 25 μg ml^−1^ concentration. It showed consistent scavenging activity with the increasing concentration, whereas the standard BHA and l-ascorbic acid exhibited 76.15 and 80.53% scavenging activity at the highest concentration of 125 μg ml^−1^, respectively.

#### 3.4.5. Reducing power

**Fig. 9:**
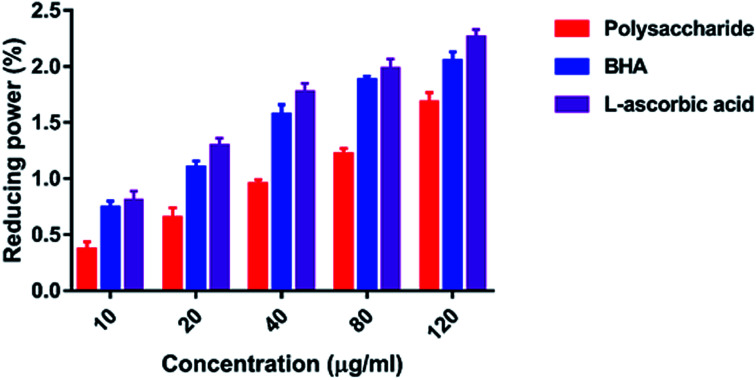
Reducing power.

The reducing capacity of a compound may serve as a significant indicator of its potent antioxidant activity, and the reducing properties are generally associated with the presence of reductions, which can break the free radical chain *via* the donation of a hydrogen atom.^47^ The polysaccharide showed the reducing power of 0.366–1.678% at 10–120 μg ml^−1^ concentration (Fig. 9). The maximum reducing power was 1.678% at 10 μg ml^−1^, and the minimum reducing power was 0.366% at 120 μg ml^−1^ concentration. Increasing the concentration of polysaccharide increases the reducing power ability. The standard BHA and l-ascorbic acid showed 2.050 and 2.260% reducing power, respectively, at 120 μg ml^−1^.

### 3.5. Cytotoxicity and anticancer activity of the polysaccharide

**Fig. 10:**
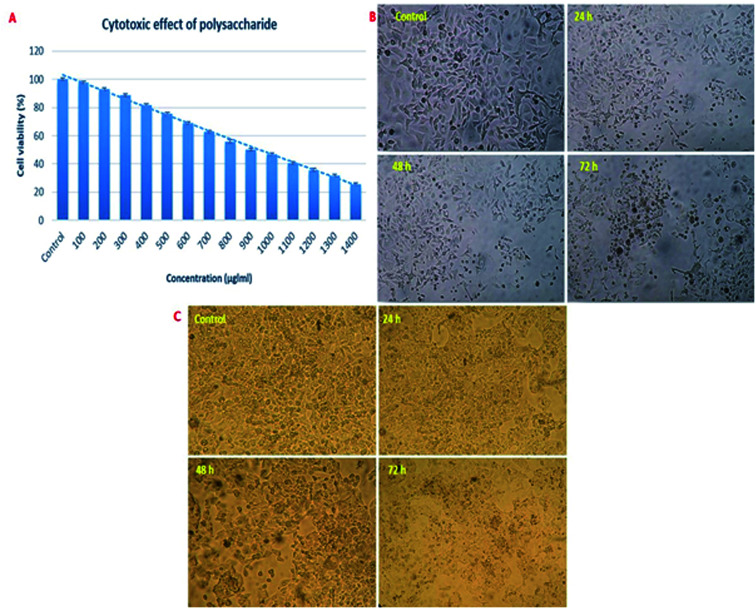
Cytotoxic effect and anticancer activity of the polysaccharide. (A) Cytotoxic effect on the Vero cell line. (B) Anticancer activity against the AGS human gastric carcinoma cell line and (C) HepG2 cell line.

For the development of anticancer drugs, initial screening of the isolated compounds should be carried out before their administration to the cancer cells to investigate the cytotoxicity effect of these compounds on normal cell lines. In the present study, the polysaccharide was initially screened for cytotoxicity effects on a normal cell line (Vero cells). The cytotoxic concentration CC_50_ value of the polysaccharide was 900 μg ml^−1^ for a 72 h incubation period (Fig. 10A). On further evaluation, the reported polysaccharide showed the anticancer activity of 18.61, 33.70, 49.65 and 63.21% against the AGS human gastric carcinoma cell line and 15.30, 29.67, 45.01 and 59.70% against the HepG2 cell line at 100, 200, 300, 400 and 500 μg ml^−1^ concentrations, respectively (Fig. 10B and C).

## 4. Discussion

The yields of the crude and fractionated polysaccharide (F1, F2 and F3) obtained from *Agaricus blazei* were 14 and 37, 37 and 14%, respectively, on a dry weight basis.^27^ Similarly, the yields of three fractionated polysaccharides (NTHSP-A, NTHSP-B and NTHSP-C) obtained from *Hohenbuehelia serotina* were 77.84%, 9.76%, and 12.40% (w/w) on a dry weight basis.^28^ In addition, the yields of the crude polysaccharide and fractionated polysaccharide (FI and FII) obtained from the edible mushroom *Lentinus sajor-caju* were 25, 14 and 9 mg, respectively.^39^ The yields of the hot water-extracted crude and purified polysaccharide from *Meripilus giganteus* were 25 and 17 mg, respectively.^30^ The variation in the yield of the polysaccharide may differ from that of the cell wall polysaccharide composition. In addition, the total carbohydrate content of the fractionated polysaccharide (G1) obtained from *Fomitiporia punctata* was 92.78 ± 2.61%.^32^ He *et al.*^33^ noted the carbohydrate and protein content of the crude and purified polysaccharide obtained from the pent mushroom *Pleurotus eryngii* as (55.22 ± 1.46% & 98.78 ± 0.12%) and (39.89 ± 1.32% & 1.15 ± 0.014%), respectively. The carbon, hydrogen and nitrogen contents of the present polysaccharide were 31.53, 4.28 and 3.01%, respectively. Similarly, the carbon, hydrogen, nitrogen and sulphur contents of the sulphated polysaccharide obtained from *Gracilaria corticata*, *Acanthophora spicifera* and *Ulvan* were 33.19–38.08%, 5.91–7.04%, 7.21–8.04% and 0.28–3.75%, respectively.^20,34,35^ Moreover, the molecular weight of glucan obtained from *Meripilus giganteus* was 1.48 × 10^5^ Da.^30^ Similarly, the two purified polysaccharides HLP1-1 and HLP2-1 obtained from the Bachu mushroom *Helvella leucopus* displayed the molecular weights of 21382 Da and 23063 Da, respectively.^38^ The variation in the molecular weight is due to the influence of the extraction method used with the solvent system.

The polysaccharides obtained *via* microwave-assisted extraction (MAE) and pressurized liquid extraction (PLE) of *Pleurotus ostreatus* and *Ganoderma lucidum* had (91.0 & 91.3%) and (89.6 & 86.5%) of glucose and (3.5 & 2.4%) and (7.1 & 9.4%) galactose, respectively. Moreover, it was reported that the mushroom extracts were rich in glucan polysaccharides recovered by the tested methods.^33^ Thus, it was speculated that the polysaccharide consisted of repeating units of terminal →6)-Glcp-(1→6), Glcp-(1→6), Glcp-(1→6), Glcp-(1→6) and Glcp-(1→ residues. Hay *et al.*^42^ reported that the GLC analysis of the alditol acetates of the periodate-oxidized, NaBH4-reduced and hydrolyzed products showed the presence of glucose units only. This good correlation between the terminal and the branched moieties and the linkages were confirmed in the technical study reported by Sims *et al.*^43^

In the present study, the DPPH radical scavenging activity of the polysaccharide was higher when compared with that of the previously studied polysaccharide obtained from the wild edible mushroom *Lentinus sajor-caju*, which was 6.14, 21.88, 30.56, 38.89 and 52.5% at 0.1, 0.5, 0.75, 1.0 and 1.5 mg ml^−1^ concentrations, respectively.^29^ He *et al.*^33^ reported the DPPH radical scavenging activity of the crude polysaccharide obtained from the pent mushroom *Pleurotus eryngii* as 85.89% at the concentration of 1.0 mg ml^−1^. In the present study, the DPPH radical scavenging activity of the polysaccharide may be due to their hydrogen donating ability. Similarly, the ABTS free radical scavenging ability of the sulfated polysaccharide obtained from *Monostroma oxyspermum* was 25.37–76.81% at various concentrations ranging from 25 to 125 mg ml^−1^.^16^ In addition, the ABTS free radical scavenging activity of the polysaccharide obtained from the Jinqian mushroom was 63.96% at the concentration of 5 mg ml^−1^.^47^ Similarly, the superoxide scavenging activity of the crude polysaccharide obtained from the pent mushroom *Pleurotus eryngii* was 85.74% at the concentration of 1.0 mg ml^−1^.^33^ Maity *et al.*^30^ reported that the superoxide radical scavenging activity of glucan obtained from *Meripilus giganteus* was 74.60% at 100 μg ml^−1^ concentration.

The hydroxyl radicals are the most harmful reactive oxygen species that can attack^46^ and damage almost every bio-macromolecule in living cells and induce severe damage to the adjacent biomolecules.^48^ Pattanayak *et al.*^29^ explained that the hydroxyl radical scavenging activity of the polysaccharide obtained from the wild edible mushroom *Lentinus sajor-caju* was 18.7, 28.5, 36.5, 43.06 and 53.3% at 0.25, 0.50, 0.75, 1.0 and 1.5 mg ml^−1^ concentration, respectively. In the present study, the hydrogen ions were released from the hydroxyl groups (–OH) of the polysaccharide and combined with radicals that terminated the radical-initiated chain reactions. In addition, the reducing power of the polysaccharide obtained from the wild edible mushroom *Lentinus sajor-caju* was 0.328, 0.443 and 0.539% at 1.0, 1.5 and 2.0 mg ml^−1^ concentrations.^29^ The reducing power of the polysaccharide may be observed in the presence of the glucopyranosyl chain reaction.

The crude and purified sulfated polysaccharide showed cytotoxicity at 1200 and 1400 μg ml^−1^ concentration on the normal cell line (HBL-100).^50^ Similarly, the two polysaccharide fractions HLP1-1 and HLP2-1 obtained from the Bachu mushroom *H. leucopus* showed the anti-proliferative activities of 26.60 and 62.19% at the concentration of 2000 μg ml^−1^ against the HepG2 cells, respectively.^49^ The active principle of the polysaccharide may enhance the level of anti-oxidation and remove the reactive oxygen species in cancer cells; thus, this polysaccharide may inhibit the cell growth.

## 5. Conclusion

The present study revealed that the polysaccharide obtained from *P. sajor-caju* had the →6)α-d-Glc^iv^(1→6)α-d-Glc^iii^(1→6)β-d-Glc^ii^(1→6)α-d-Glc^i^(1→structure; glucose linkages, for example, β-d-Glc^ii^(1→6) linkages, in the fundamental chain of the glucan and branch moieties are required for the anticancer action. At the molecular level, the improvement of the anticancer action of polysaccharides and their clinical characteristics by expanding their water solubility and capacity to enter the gastrointestinal wall after administration is regularly conducted; carboxymethylation is one of the principal techniques that the body uses for chemical interactions in anticancer activity; this is clear from this study. Therefore, the active principle of the polysaccharide may be helpful in preventing oxidative damage in human cells and enhancing the level of antioxidants to prevent the formation of reactive oxygen species, which inhibits the cancer cells. Hence, the mushroom polysaccharide might be used as a futuristic food and functional compound in the pharmacological industry.

## Conflicts of interest

All authors declare that there is no conflict of interest.

## Supplementary Material
